# Dynamic phenotypic shifts and M2 receptor downregulation in bladder smooth muscle cells induced by mirabegron

**DOI:** 10.3389/fphar.2024.1446831

**Published:** 2024-07-24

**Authors:** A. E. Muderrisoglu, A. Ciotkowska, B. Rutz, S. Hu, S. Qian, A. Tamalunas, C. G. Stief, M. Hennenberg

**Affiliations:** ^1^ Department of Urology, LMU University Hospital, LMU Munich, Munich, Germany; ^2^ Department of Medical Pharmacology, Istanbul Medipol University, Istanbul, Türkiye

**Keywords:** overactive bladder (OAB), lower urinary tract symptoms (LUTS), storage symptoms, mirabegron, β3-adrenoceptor, bladder smooth muscle contraction, detrusor overactivity, phenotype plasticity

## Abstract

**Introduction:**

Mirabegron is available for treatment of overactive bladder (OAB). However, mechanisms underlying symptom improvements and long-term effects on bladder smooth muscle cells are uncertain. Contractility and growth of bladder smooth muscle contribute to OAB, and depend on smooth muscle phenotypes, and on muscarinic receptor expression. Here, we examined prolonged exposure to mirabegron (20–48 h) on phenotype markers, muscarinic receptor expression, and phenotype-dependent functions in human bladder smooth muscle cells (hBSMC).

**Methods:**

Expression of markers for contractile (calponin, MYH11) and proliferative (MYH10, vimentin) phenotypes, proliferation (Ki-67), and of muscarinic receptors were assessed by RT-PCR. Proliferation, viability, actin organization and contractions in cultured hBSMC were examined by EdU, CCK-8, phalloidin staining and matrix contraction assays.

**Results:**

Calponin-1 mRNA decreased with 100 nM and 150 nM mirabegron applied for 20 h (0.56–0.6 fold of controls). Decreases were resistant to the β_3_-AR antagonist L-748,337 (0.34–0.55 fold, 100–150 nM, 20 h). After 40 h, decreases occured in the presence of L-748,337, but not without L-748,337. MYH11 mRNA increased with 150 nM mirabegron (40 h, 1.9 fold). This was partly preserved with L-748,337, but not observed after 20 h mirabegron exposure. Vimentin mRNA reduced with 150 nM mirabegron after 20 h, but not after 40 h, with and without L-748,337 (0.71–0.63 fold). MYH10 mRNA expression remained unaffected by mirabegron. Exposure to 150 nM mirabegron increased Ki-67 mRNA after 20 h in the presence of, but not without L-748,337, and after 40 h without, but not with L-748,337. Proliferation rates and actin organization were stable with 50–150 nM mirabegron (24 h, 48 h). Viability increased significantly after mirabegron exposure for 20 h, and by trend after 40 h, which was fully sensitive to L-748,337. M2 mRNA was reduced by 20 h mirabegron, which was resistant to L-748,337. Carbachol (3 µM) enhanced time-dependent contractions of hBSMC, which was inhibited by mirabegron (150 nM) in late phases (24 h), but not in early phases of contractions.

**Conclusion:** Mirabegron induces dynamic phenotype alterations and M2 downregulation in hBSMC, which is paralleled by time-shifted anticontractile effects. Phenotype transitions may be involved in improvements of storage symptoms in OAB by mirabegron.

## 1 Introduction

Mirabegron, a β_3_-adrenoceptor (β_3_-AR) agonist, is used for treatment of storage symptoms in overactive bladder (OAB) (Nambiar et al., 2018; Igawa et al., 2019; Gravas et al., 2023). In healthy conditions, voiding occurs by detrusor contraction following activation of muscarinic receptors on bladder smooth muscle cells, while this mechanism is overactivated and causes storage symptoms in OAB (Michel et al., 2023). Muscarinic antagonists improve storage symptoms and are the first-line option for medical treatment of OAB, while long term adherence is low due to unmet expectations of patients to this drug class along with unbalanced side effects (Yeowell et al., 2018; Muderrisoglu et al., 2022). Mirabegron has been introduced as an alternative and may be effective in patients showing insufficient improvements with antimuscarinics or not tolerating them (Oelke et al., 2013; Michel et al., 2023). While it was initially believed that mirabegron improves symptoms by relaxing bladder smooth muscle via β_3_-AR, this concept has been challenged following approval (Igawa et al., 2019; Huang et al., 2022). In fact, relationships between mirabegron, smooth muscle phenotype and muscarinic receptors are still unclear. Available *in vitro* studies with mirabegron are limited to its acute effects occuring within minutes or few hours, but did not yet include prolonged exposure in the range of days with physiologic concentrations of mirabegron, i.e., conditions analog to chronic treatment.

In general, phenotype plasticity of smooth muscle cells may determine their contractility, and includes a contractile and a non-contractile, so-called synthetic phenotype characterized by weak contractility but high proliferation. Transitions between both phenotypes are possible. Contractile properties depend on expression of different isoforms of smooth muscle myosin heavy and light chains (Babu et al., 2006). Smooth muscle myosin heavy chain 11 (MYH11) is an abundant cytoskeletal protein in smooth muscle cells of various organs including vessels, intestine, bladder and uterus (Yokoyama et al., 2018). From four investigated MYH11 isoforms, including SM-1, SM-2, SM-A and SM-B, SM-B mRNA expression appeared most robust in the bladder, and is required for force generation and rapid voiding (Low et al., 2006; Li et al., 2018; Yokoyama et al., 2018). However, loss of one, several or all isoforms affects the contractile function of bladder smooth muscle (Low et al., 2006; Li et al., 2018). Myosin heavy chain 10 (MYH10) is one of several isoforms of non muscle myosin II, also known as non-muscle myosin heavy chain B, and has been detected in smooth muscle cells during proliferation and under some pathological conditions (Sjuve et al., 2001; Obara et al., 2005; Hong et al., 2015). MYH10 is involved in cell migration (Hong et al., 2015) and downregulated in smooth muscle cells paralleled by growth inhibition (Obara et al., 2005). Calponin is an actin filament-associated regulatory protein primarily found in contractile smooth muscle (Feng et al., 2019). Vimentin is expressed in the undifferentiated and proliferative cells of mesenchymal origin, and is used as a synthetic phenotype marker to determine phenotypic switches of human vascular smooth muscle cells from contractile to synthetic (Javed et al., 2020; Zhang et al., 2021; Balint et al., 2023). However, intermediate filaments, including desmin and vimentin, have been proposed to be coupled to contractile units through dense bodies (Javed et al., 2020). In a study using an animal model, vimentin was suggested to required for active force development in airway smooth muscles (Wang et al., 2007). Together, phenotype transformations are characterized by changes in expression of smooth muscle markers, such as myosin heavy chains, calponin and vimentin, which are used as markers for phenotype identification of smooth muscle cells (Zhong et al., 2018).

In parallel, dynamic phenotype plasticity is known from bladder pathologies, at organ and cellular levels, including initial phases of bladder outlet obstruction (BOO), where transient decompensation mechanisms initiate molecular events leading to hypertrophy of detrusor smooth muscle cells (Michel et al., 2023). The ability of smooth muscle to compensate for increased functional demands is associated with alterations in the expression of proteins in the thick and thin filaments (Mannikarottu et al., 2006). Although these transient mechanisms are known from several studies, and even though impacts on phenotype regulation may explain symptom improvements in OAB, effects of mirabegron on phenotype plasticity of human bladder smooth muscle cells (i.e., contractile vs. non-contractile phenotype) have not yet been addressed. Similarly, effects of mirabegron on muscarinic receptors have been examined in functional studies with acute administration of mirabegron, while effects of long-term exposure of muscarinic receptors in the bladder to mirabegron have not been reported.

Here, we examined effects of exposure to mirabegron for up to 2 days on phenotype markers, on muscarinic receptor expressions in human bladder smooth muscle cells (hBSMC), and on phenotype-dependent functions including cell viability, proliferation, actin organization and contractility of hBSMC.

## 2 Materials and methods

### 2.1 Cell culture

Human bladder smooth muscle cells (hBSMC) were obtained from Sciencell Research Laboratories (Carlsbad, CA, United States) (catalog #4310, lot #5828). Information about the sex of donors was not provided, and not available upon request. Cells were cultured in smooth muscle cell medium (SMCM) (Sciencell Research Laboratories) containing fetal bovine serum (FBS, 2%), smooth muscle cell growth supplement (1%) and penicillin/streptomycin (1%). When the cells reached 90% confluency, they were passaged for the next experiments, so that cells from 1 to 10 passages were used for experiments in this study. Before treating cells with mirabegron (50, 100, 150 nM) or DMSO (solvent of applied drugs, for controls; always 0.1% of medium), the medium was changed by FBS-free medium. Subsequently, cells were incubated at 37°C with 5% CO_2_ during treatment periods (20, 40 h). To assess β_3_-AR-independent effects of mirabegron, experiments with mirabegron were repeated in the presence of the β_3_-AR antagonist L-748,337 (1 µM). L-748,337 was added to hBSMC in FBS-free medium 1.5 h before addition of mirabegron.

### 2.2 RT-PCR

hBSMC were seeded with a density of 300,000 cells/well in 6-well plates. After 24 h, cells were treated with mirabegron, DMSO and/or L-748,337 for indicated periods. RNA was isolated using the RNeasy Mini Kit (Qiagen, Hilden, Germany) based on the manufacturer’s instructions. RNA concentrations were measured spectrophotometrically. cDNA was synthesized using 500 ng of isolated RNA using a Reverse Transcription System (Promega, Madison, WI, United States). Real-time PCR (RT-PCR) was performed with a Roche Light Cycler (Roche, Basel, Switzerland). Primers (with RefSeq accession no) for calponin-1 (CNN1, NM_001299), myosin heavy chain 11 (MYH11, NM_022844), myosin heavy chain 10 (MYH10, NM_005964), vimentin (VIM, NM_003380), Ki67 (NM_001145966), cholinergic receptor muscarinic 3 (CHRM3, NM_000740), muscarinic 2 (CHRM2, NM_000739), and glyceraldehyde-3-phosphate dehydrogenase for housekeeping (GAPDH, NM_002046) were purchased from Qiagen (Hilden, Germany). PCR reaction volume was 10 μL, which included 5 µL FastStart DNA MasterPlus SYBR Green I (Roche, Basel, Switzerland), 1 μL primer, 1.5 μL PCR grade water and 2.5 μL sample. Denaturation was performed at 95°C for 10 min, and amplification with 40 cycles, each including 10 s at 95°C, 10 s at 60°C, 15 s at 72°C. PCR product quality was demonstrated by post-PCR melt curve analysis. All samples were determined in duplicate and presented as means of these two replicates. Results were expressed using the ΔΔCt method, where number of cycles (Ct) at which the fluorescence signal exceeded a defined threshold for GAPDH (see Supplementary Figure S1 for Ct values for GAPDH) was subtracted from Ct values for targets (Ct_target_-Ct_GAPDH_ = ΔCt), and values were calculated as 2^−ΔΔCT^ and normalized to the mean values of corresponding controls.

### 2.3 Western blot

For protein isolation, cells were washed twice with ice-cold phosphate-buffered saline (PBS). 400 μL of radioimmunoprecipitation assay (RIPA) buffer (Sigma-Aldrich, Munich, Germany) containing 1% protease inhibitor cocktail (P8340) (Sigma-Aldrich, Munich, Germany) were added to each flask. After keeping on ice for 1 min, cells were scraped from flasks. Lyzed cell suspensions were incubated on ice for 10 min, vortexed each 2 min, and centrifuged (10,000 rpm, 5 min, 4°C). 5 μL of each sample was used for protein determination, using a protein quantification assay kit (catalog #740967, Macherey-Nagel, Düren, Germany) according to the manufacturer’s instructions. Remaining samples were boiled for 10 min with laemmli sample buffer (1610737) (Bio-Rad, Hercules, CA, United States) containing 5% of beta-mercaptoethanol (M6250) (Sigma-Aldrich, Munich, Germany). Samples (30 μg/lane) were subjected to SDS polyacrylamide gel electrophoresis, and proteins were blotted on Protran^®^ nitrocellulose membranes (Cytiva 10600008, Sigma-Aldrich, Munich, Germany). For blockade of unspecific binding sites, membranes were blocked with PBS containing 5% milk powder (Roth, Karlsruhe, Germany) over night, and incubated with mouse monoclonal anti β-actin antibody (sc-47778, 1:500) (Santa Cruz Biotechnology, Santa Cruz, CA, United States), mouse monoclonal anti calponin1/2/3 antibody (sc-136987, 1:500) (Santa Cruz Biotechnology, Santa Cruz, CA, United States), or mouse monoclonal anti vimentin antibody (5G3F10, 1:1000) (Cell Signaling Technology, Beverly, MA, United States). All primary antibodies were diluted in PBS containing 0.1% Tween 20 (PBS-T) and 5% milk powder. Subsequently, detection was continued using secondary goat anti mouse IgG, H&L Chain Specific Peroxidase Conjugate (401,215) (Sigma-Aldrich, Munich, Germany) by dilutions 1:3000, 1:3000 and 1:2000, respectively. Membranes were washed with PBS-T after any incubation with primary or secondary antibodies. Blots were developed with SuperSignal west pico plus chemiluminescent substrate (34,577) (ThermoScientific, Rockford, United States) and imaged using the ChemiDoc XRS + System (Bio-Rad, Hercules, CA, United States). Intensities of resulting bands for β-actin, calponin and vimentin were quantified densitometrically using ImageJ (National Institutes of Health, Bethesda, Maryland, United States), and bands were normalized to β-actin in corresponding samples.

### 2.4 Viability assay

Cell Counting Kit-8 (CCK-8) (Sigma Aldrich, Munich, Germany) was used to assess the cell viability. 5,000 cells were seeded to each well of 96-well plates followed by culture for 24 h, before mirabegron, DMSO and/or L-748,337 were added to corresponding wells. After 20 or 40 h treatment periods, 10 μL of [2-(2-methoxy-4-nitrophenyl)-3-(4-nitrophenyl)-5-(2,4-disulfophenyl)-2H-tetrazolium monosodium salt (WST-8) from the kit were added to each well and cells were incubated for 2 h at 37°C. Finally, absorbance of each well was measured at 450 nm.

### 2.5 Proliferation assay

Proliferation rate of cells was assessed using the 5-ethynyl-2′-deoxyuridine (EdU) based EdU-Click 555 proliferation assay kit (Baseclick, Tutzing, Germany), which was applied according to the manufacturer’s instructions. hBSMC were seeded at densities of 40,000 cells/well into 12 well-chambered coverslips and cultured for 24 h. Subsequently, mirabegron or DMSO were added to corresponding wells for indicated treatment periods (24, 48 h). The medium was replaced by 10 mM EdU solution in FBS-free smooth muscle cell medium containing mentioned drugs, 24 h before the cell fixation. Cells were fixed by using 3.7% formaldehyde. EdU incorporated into DNA was assessed by detection with fluorescing 5-carboxytetramethylrhodamine (5-TAMRA). DAPI (4′,6-diamidino-2-phenylindole) was used for counterstaining of all nuclei. Cells were analyzed by fluorescence microscopy (Leica LASX, Wetzlar, Germany). The number of proliferating cells (i.e., EdU-stained cells) was calculated by using ImageJ cell counter (National Institutes of Health, Bethesda, Maryland, United States).

### 2.6 Phalloidin staining

hBSMC were plated into 12 well-chambered coverslips (40,000 cells/well) and cultured for 24 h. After treatment with mirabegron or DMSO for 24 or 48 h, staining was performed with 100 μM fluoresceine isothiocyanate- (FITC-) labelled phalloidin (Sigma-Aldrich, Munich, Germany), according to the manufacturer’s instruction. DAPI was used for counterstaining of all nuclei. Labelled cells were analyzed using a laser scanning microscope (Leica LASX, Wetzlar, Germany). Finally, staining was calculated by using ImageJ (National Institutes of Health, Bethesda, Maryland, United States).

### 2.7 Contraction assay

Contractility of cells was measured using the CytoSelect 24-Well Cell Contraction Assay Kit (Floating Matrix Model) (Cell Biolabs, San Diego, CA). hBSMC were cultured in flasks for 72 h before being trypsinized and resuspended in fresh SMCM to a dilution of 1,000,000 cells/mL. 400 μL of collagen gel working solution, which is provided with the kit and 100 µL of the trypsinized cell suspension were mixed before transferring to each well of the 24-well plate, provided with the kit as well. Subsequently, plates were incubated for 1 h (37°C, 5% CO_2_) for collagen polymerization. After 1 h of incubation, 1 mL of SMCM with or without mirabegron (150 nM) and carbachol (3 µM) were added and incubation was continued. For monitoring of collagen contraction, pictures were taken 10, 20, 30, 60, 120 min after adding of SMCM with related agents, or 6, 24, 48 h after adding SMCM with related agents. Diameters and areas of the collagen plugs and wells on pictures were quantified using ImageJ (National Institutes of Health, Bethesda, Maryland, United States), and contractions were calculated as diameter_(well)_ - diameter_(plug)_ (in cm; at indicated time points), with the well diameter (1.5 cm) representing the tension at the time point zero (i.e., when SMCM and related agents were added to wells).

### 2.8 Drugs and nomenclature

Mirabegron (2-Amino-N-[4-[2-[[(2R)-2-hydroxy-2-phenylethyl]amino]ethyl]phenyl]-4-thiazoleacetamide) is a β_3_-AR agonist, which is used for medical treatment of OAB. Binding constants (K_i_ values) of mirabegron amounted to 2.5 nM for β_3_-, 383 nM for β_1_-, and 977 nM for β_2_-adrenoceptors in radioligand assays, performed in cells transfected with human β-adrenoceptors (Tasler et al., 2012). K_i_ values for α_1_-adrenoceptors were assessed as well, in competition assays using membranes from cells transfected with human α_1_-adrenoceptors, where they amounted to 0.437 µM for α_1A_-, 1.8 µM for α_1D_-, and 26 µM for α_1B_-adrenoceptors (Alexandre et al., 2016). Antagonism in functional contraction experiments with intact tissues requires micromolar concentrations (Alexandre et al., 2016; Huang et al., 2021). Using 50 mg/daily and multiple doses, maximum plasma levels do not exceed 167 nM in healthy women and 137 nM in healthy men, with maximum concentrations reached within 3–4 h and steady state concentrations ranging lower (Krauwinkel et al., 2012). Accordingly, concentrations from 50 to 150 nM, applied in our study are probably specific for β_3_, and within the ranges of plasma concentrations. L-748,337 (N-[[3-[(2S)-2-Hydroxy-3-[[2-[4-[(phenylsulfonyl)amino]phenyl]ethyl]amino]propoxy]phenyl]methyl]-acetamide), is a β-AR antagonist, with affinity values of 4, 204, 390 nM to human β_3_-AR, β_2_-AR, β_1_-AR, respectively (Candelore et al., 1999). Both mirabegron and L-748,337 were purchased from Tocris (Bristol, United Kingdom). They were dissolved in dimethylsulfoxide (DMSO) as 10 mM stock solutions. Aliquots of stock solutions were stored −20°C until use.

### 2.9 Statistical analysis

Data analyses were performed using Graphpad Prism (version 10.0.1, GraphPad Software Inc., San Diego, CA, United States). Each series was pre-planned to contain five single experiments. However, Western blot analyses were based on seven individual experiments, because we assumed in advance that detection may typically fail in some samples. As all experiments with successfull detection were included to analysis, these results are based on more than five experiments. This procedure is in line with the explorative character of this study (Michel et al., 2020). To compare groups treated with different mirabegron concentrations versus controls (solvent-treated), one-way ANOVA or Kruskal Wallis test were performed, depending on data distribution. If the data showed Gaussian distribution, ordinary one-way ANOVA was used followed by Dunnett’s multiple comparisons test. Otherwise, i.e., if the data distribution was non-Gaussian, Kruskal Wallis test was performed followed by Dunn’s multiple comparisons test. To compare treated groups vs. the control group at each time point in contraction assays, linear regression of two-way ANOVA was used followed by Dunnett’s multiple comparison test. Data in scatter plots include values from all independent experiment (with each single value from an independent experiment reflecting means of double or triple determination), together with means. Data in diagrams showing contractions over time are means with standard deviations (SD). *P* values < 0.05 were considered statistically significant. Since our study shows an expolatory character, *p* values need to be considered as descriptive, instead of hypothesis-testing (Michel et al., 2020). In line with the recent recommendations (Michel et al., 2020), *p* values were used sparingly, and relevant effect sizes were reported as folds of means of corresponding controls with 95% confidence intervals (CI) in the text, which were calculated using Graphpad Prism. No data were excluded from each analysis. Due to technical reasons, incubation times differed between experiments including mRNA detection (20, 40 h) and functional experiments (24, 48 h). Initially, incubation for 24–48 h was planned for RT-PCR samples as well, but detection completely failed in these samples, for unknown (probably technical) reasons. Consequently, new samples with different, but similar incubation periods were prepared and analyzed.

## 3 Results

### 3.1 Effects of mirabegron on contractile phenotype markers

**FIGURE 1:**
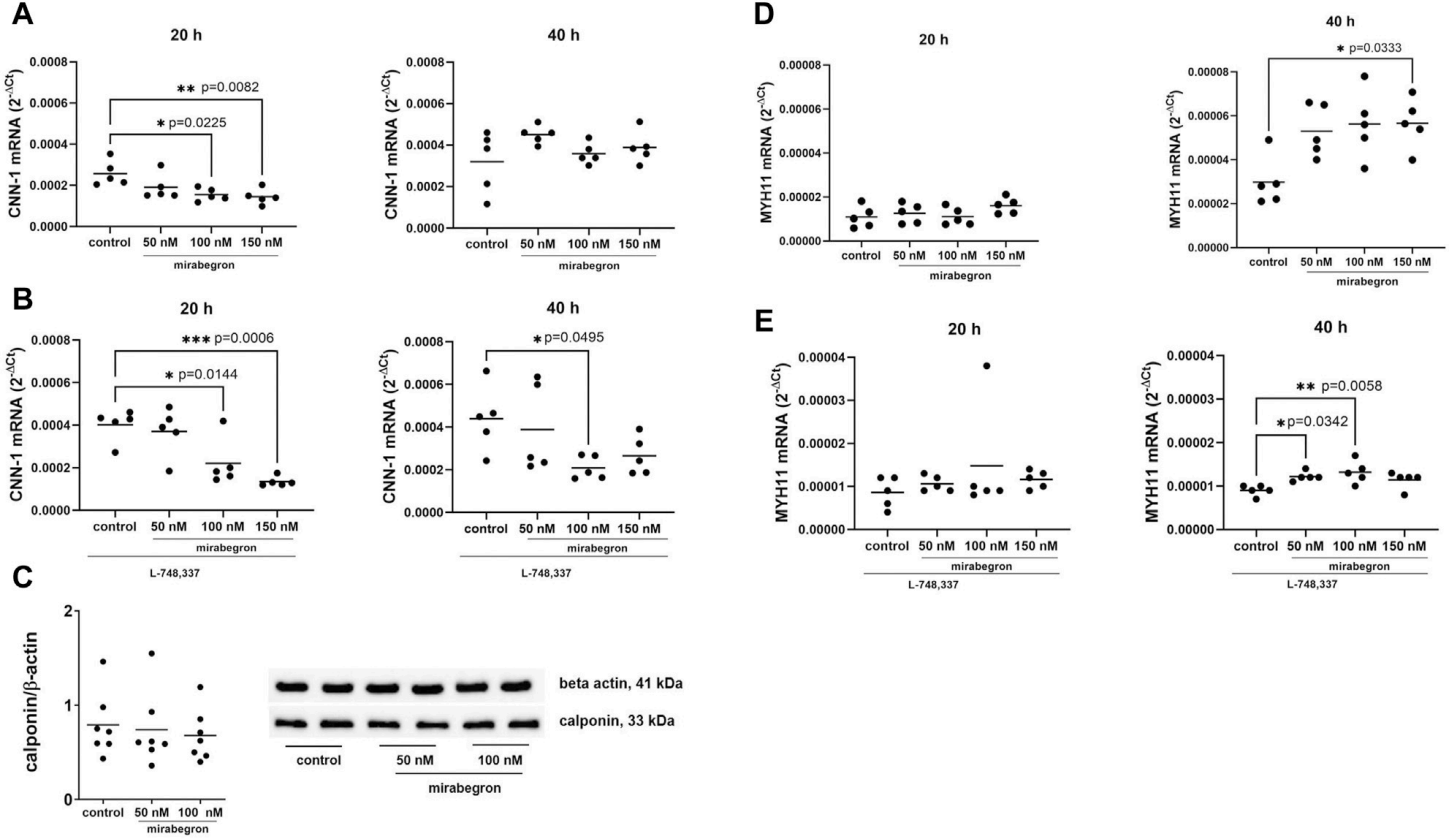
Effects of mirabegron on contractile phenotype markers in hBSMC. mRNA expressions of calponin-1 (CNN-1) **(A)**, calponin-1 in the presence of L-748,337 (1 µM) **(B)**, myosin heavy chain 11 (MYH11) **(D)** and myosin heavy chain 11 in the presence of L-748,337 (1 µM) **(E)** were determined by RT-PCR after 20 h or 40 h of mirabegron exposure or after analog treatment with solvent (controls). Protein expressions of calponin-1 are shown as ratios of band intensities, together with representative Western blots **(C)**. Shown are all data from n = 5 independent experiments for RT-PCR, and n = 7 independent experiments for Western blot (see Supplementary Figure S2 for blots from each experiment), together with means, and with *p* values from Kruskal Wallis followed by Dunn’s multiple comparisons test **(A,D)** and ordinary one-way ANOVA followed by Dunnett’s multiple comparisons test **(B,C,E)** (*p < 0.05, **p < 0.01, ***p < 0.001 vs. control).

Calponin-1 mRNA expression decreased with 100 nM and 150 nM of mirabegron, applied for 20 h. Values for mRNA expression amounted to 0.74 fold (0.43, 1.04) of mean of controls with 50 nM, 0.6 fold (0.45, 0.75) with 100 nM (*p* = 0.0225) and 0.56 fold (0.38, 0.74) with 150 nM (*p* = 0.0082). No reductions were observed after 40 h, where values mounted to 1.41 fold (1.24, 1.58) of mean of controls with 50 nM, 1.12 fold (0.92, 1.32) with 100 nM and 1.22 fold (0.92, 1.52) with 150 nM (Figure 1A).

With preceding addition of L-748,337 (1 µM), mRNA expression of calponin-1 reduced with mirabegron after both 20 and 40 h. Referred to means of controls, calponin-1 mRNA levels amounted to 0.92 fold (0.57, 1.27) with 50 nM, 0.55 fold (0.2, 0.9) of with 100 nM (*p* = 0.0144) and 0.34 fold (0.26, 0.41) with 150 nM (*p* = 0.0006) after 20 h, and to 0.89 fold (0.3, 1.48) with 50 nM, 0.48 fold (0.32, 0.63) with 100 nM (*p* = 0.0495) and 0.6 fold (0.35, 0.86) with 150 nM after 40 h (Figure 1B).

Protein expression of calponin-1 did not differ between groups (Figure 1C; Supplementary Figure S2) after 24 h. Mean ratios of band intensities for calponin-1 and beta actin mounted to 0.79 (0.47, 1.1) for controls, 0.74 (0.37, 1.1) with 50 nM, and 0.68 (0.42, 0.93) with 100 nM mirabegron.

In contrast, expression of MYH11 mRNA increased with 150 nM of mirabegron applied for 40 h, while it remained unchanged after 20 h. Referred to means of controls, increases amounted to 1.78 fold (1.28, 2.28) with 50 nM, 1.89 fold (1.24, 2.53) with 100 nM, and 1.9 fold (1.43, 2.38) with 150 nM (*p* = 0.0333) after 40 h (Figure 1D).

Increases were preserved in the presence of L-748,337. Values amounted to 1.21 fold (0.93, 1.49) of means of controls with 50 nM, 1.73 fold (−0.13, 3.59) with 100 nM and 1.34 fold (1.07, 1.62) with 150 nM after 20 h, and to 1.36 fold (1.2, 1.5) with 50 nM (*p* = 0.0342), 1.45 fold (1.13, 1.78) with 100 nM (*p* = 0.0058) and 1.25 fold (1.01, 1.49) with 150 nM after 40 h (Figure 1E).

### 3.2 Effects of mirabegron on non-contractile phenotype markers

**FIGURE 2:**
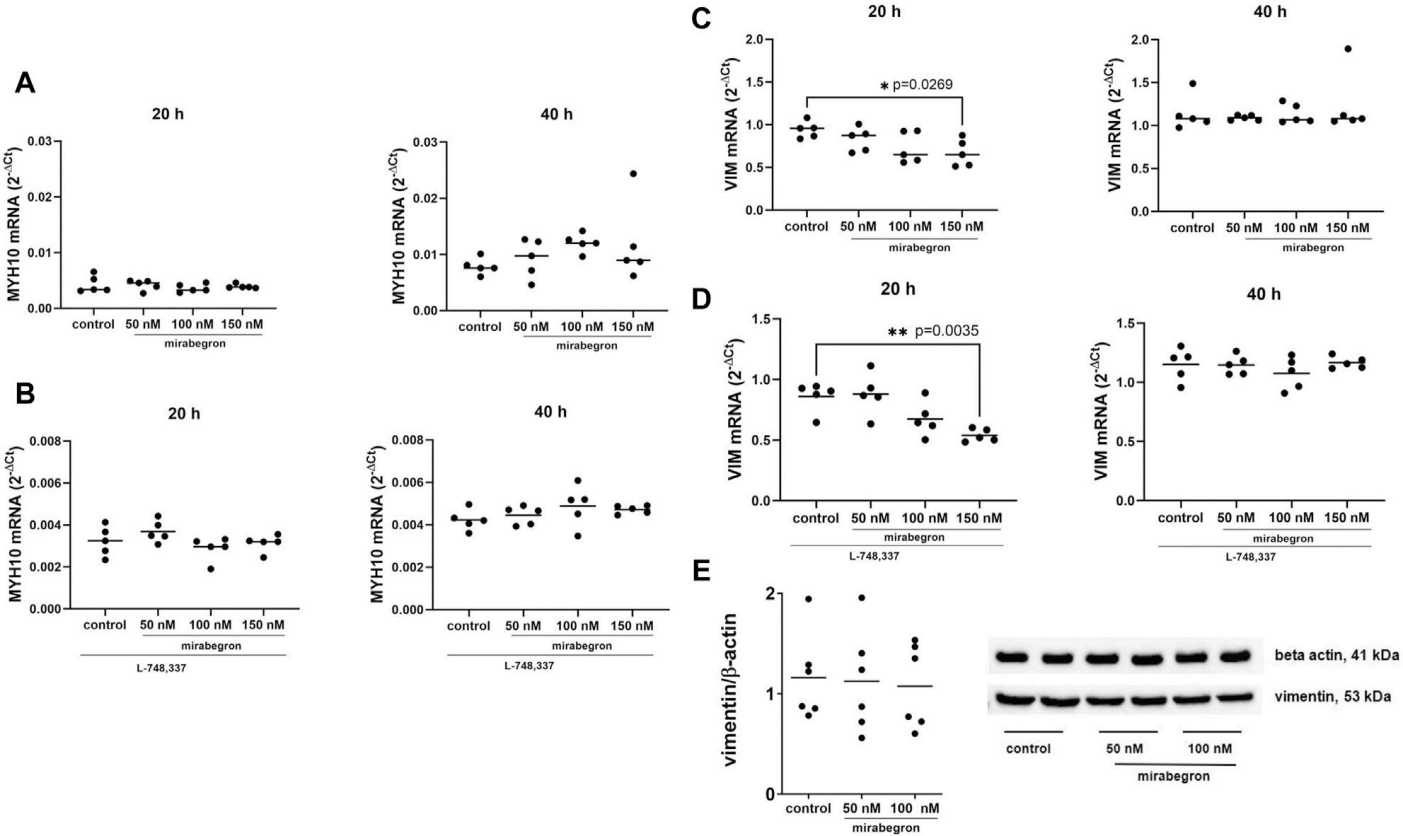
Effects of mirabegron on non-contractile phenotype markers in hBSMC. mRNA expressions of myosin heavy chain 10 (MYH10) **(A)**, myosin heavy chain 10 in the presence of L-748,337 (1 µM) **(B)**, vimentin (VIM) **(C)** and vimentin in the presence of L-748,337 (1 µM) **(D)** were determined by RT-PCR after 20 h or 40 h of mirabegron exposure or after analog treatment with solvent (controls). Protein expressions of vimentin are shown as ratios of band intensities, together with representative Western blots **(E)**. Shown are all data from n = 5 independent experiments for RT-PCR, and n = 6 independent experiments for Western blot (see Supplementary Figure S2 for blots from each experiment), together with means, and with *p* values from Kruskal Wallis followed by Dunn’s multiple comparisons test **(A)** and ordinary one-way ANOVA followed by Dunnett’s multiple comparisons test **(B–E)** (*p < 0.05, **p < 0.01 vs. control).

MYH10 mRNA expression was mostly unchanged in mirabegron-treated groups, both with or without L-748,337 (Figures 2A, B). Slight increases were observed after 40 h, amounting to 1.18 fold (0.64, 1.72) of mean of controls with 50 nM, 1.53 fold (1.27, 1.79) with 100 nM, and 1.51 fold (0.38, 2.65) with 150 nM (Figure 2A).

In contrast, vimentin mRNA expression reduced with 150 nM mirabegron after 20 h, both with or without L-748,337, but was stable after 40 h among all groups. Referred to mean of controls, vimentin mRNA mounted to 0.88 fold (0.7, 1.06) with 50 nM, 0.77 fold (0.53, 1.02) with 100 nM, and 0.71 fold (0.5, 0.92) with 150 nM (*p* = 0.0269) (Figure 2C). With L-748,337, they amounted to 1.02 fold (0.77, 1.27) of means of controls with 50 nM, 0.79 fold (0.58, 0.99) with 100 nM, and 0.63 fold (0.55, 0.7) with 150 nM (*p* = 0.035) (Figure 2D).

Protein expression of vimentin was similar among groups (Figure 2E; Supplementary Figure S2). The mean ratios of band intensities for vimentin and β-actin were 1.16 (0.7, 1.62) for controls, 1.12 (0.58, 1.67) with 50 nM, and 1.08 (0.63, 1.51) with 100 nM mirabegron after 24 h.

### 3.3 Effects of mirabegron on proliferation

**FIGURE 3:**
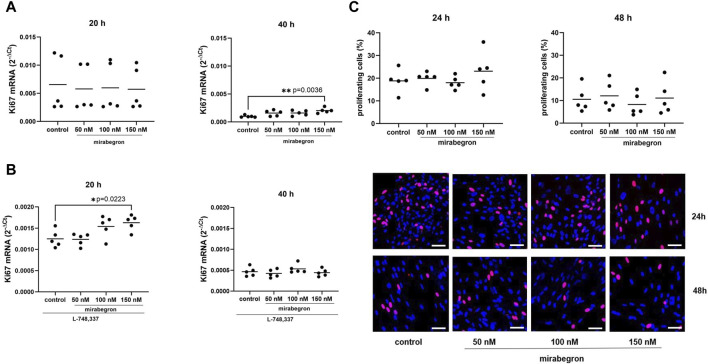
Effects of mirabegron on proliferation in hBSMC. mRNA expressions of Ki-67 **(A)** and of Ki-67 in the presence of L-748,337 (1 µM) **(B)** were determined by RT-PCR after 20 h or 40 h of mirabegron exposure or after analog treatment with solvent (controls). Proliferation was assessed by EdU assays (red, proliferating cells; blue, nonproliferating cells; scale bars: 50 µm), after treatment with mirabegron or solvent (controls) as indicated **(C)**. Shown are all data from n = 5 independent experiments, together with means, and with *p* values from Kruskal Wallis followed by Dunn’s multiple comparisons test (**A**, 20 h; **C**, 48 h) and ordinary one-way ANOVA followed by Dunnett’s multiple comparisons test (**A,** 40 h; **B,C**, 24 h) (*p < 0.05, **p < 0.01 vs. control).

Expression of Ki67 mRNA was mostly stable in groups treated for 20 h with mirabegron, but increased to 1.6 fold (0.95, 2.25) of mean of controls with 50 nM, 1.6 fold (1.03, 2.2) with 100 nM and 2.1 fold (1.5, 2.6) with 150 nM (*p* = 0.036) after 40 h (Figure 3A). However, increases after exposure to mirabegron for 20 h were observed in the presence of L-748,337, mounting to 0.99 fold (0.85, 1.13) of mean of controls with 50 nM, 1.23 fold (0.98, 1.49) with 100 nM and 1.3 fold (1.12, 1.49) with 150 nM (*p* = 0.0223) (Figure 3B).

In contrast, proliferation rates assessed by EdU assays were similar among groups, on average reaching 19% (12.5, 25.1) in controls, and 20% (16.2, 23.7), 18% (14.6, 21.5) and 23% (12.4, 33.8) with 50 nM, 100 nM and 150 nM of mirabegron after 24 h. After 48 h, average proliferation rates reached 11% (3.5, 17.5) in controls, and 12% (4.1, 20.0), 8% (2.2, 14.3) and 11% (2.1, 20.2) with 50 nM, 100 nM and 150 nM of mirabegron (Figure 3C).

### 3.4 Effects of mirabegron on actin organization

**FIGURE 4:**
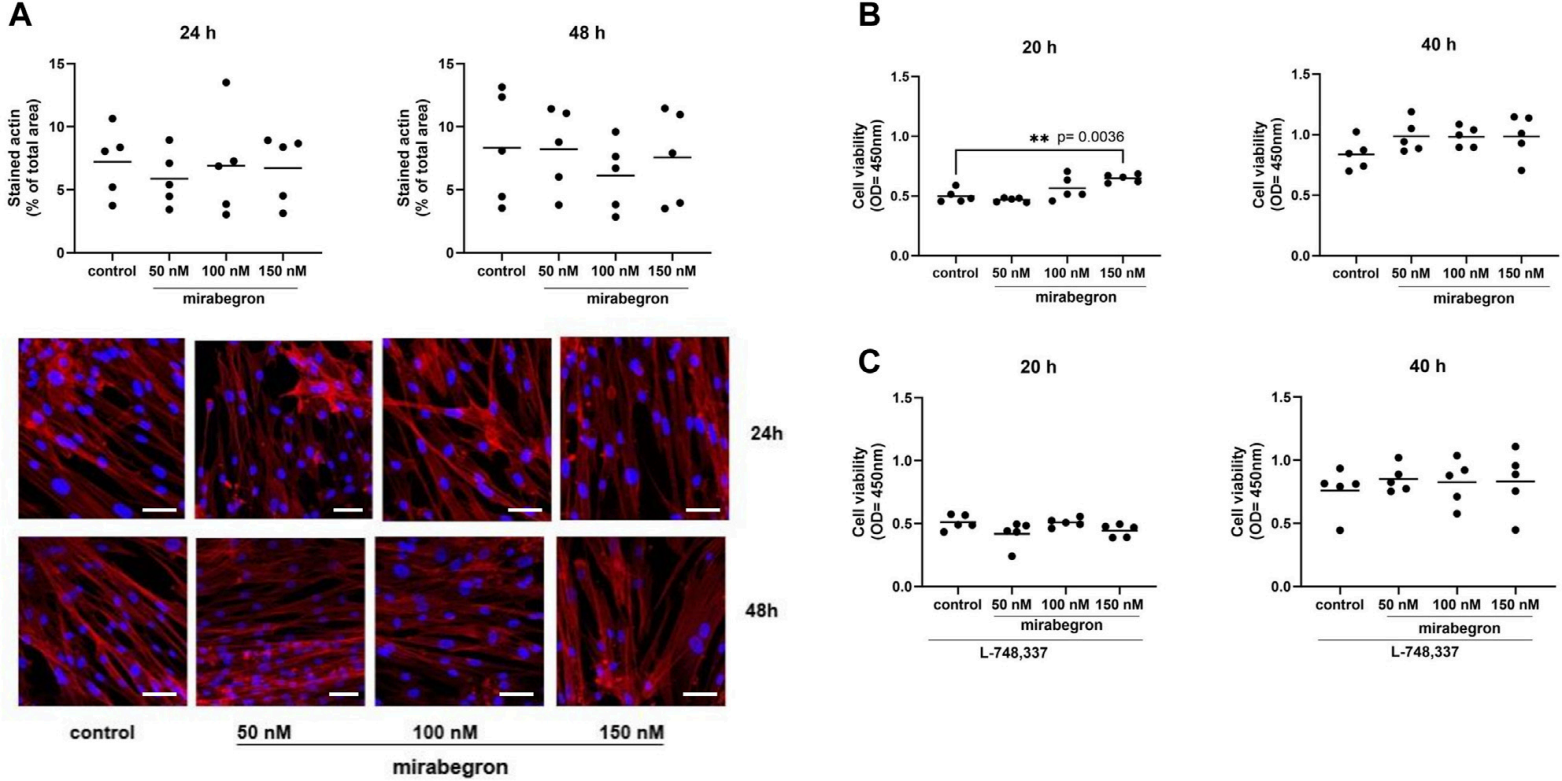
Effects of mirabegron on actin organization and cell viability in hBSMC. Actin organization of cells was assesed by phalloidin staining, after treatment with mirabegron or solvent (controls) as indicated. Actin polymers were stained by phalloidin (red), nuclei were counterstained with DAPI (blue) (scale bars: 50 µm) **(A)**. Viability was assessed by CCK-8 assay, after 20 h or 40 h of mirabegron exposure in absence **(B)** or presence **(C)** of L-748,337 (1 µM), or after analog treatment with solvent for controls. Shown are all data from n = 5 independent experiments, together with means, and with *p* values from Kruskal Wallis followed by Dunn’s multiple comparisons test (**A,C**, 40 h) and ordinary one-way ANOVA followed by Dunnett’s multiple comparisons test (**B,C**, 20 h) (**p < 0.01 vs. control).

All control and mirabegron-treated groups were characterized by distinct and visible actin filaments, visualized by phalloidin staining (Figure 4A). Actin in these cells was organized to long filaments and bundles, showing parallel arrangement and shared orientation. Percentages of phalloidin-stained actin areas were similar with all concentrations and both exposure durations. Average phalloidin-stained areas reached 7.2% (3.8, 10.6) in controls and 5.9% (3.2, 8.6), 7% (1.8, 12.0) and 6.7% (3.4, 10.0) with 50 nM, 100 nM and 150 nM of mirabegron, after 24 h. Similarly, average phalloidin-stained areas reached 8.3% (2.9, 13.8) in controls and 8.2% (4.1, 12.3), 6.1% (2.7, 9.6) and 7.6% (2.9, 12.2) with 50 nM, 100 nM and 150 nM of mirabegron, after 48 h.

### 3.5 Effects of mirabegron on cell viability

Cell viability was increased by 16% (−22.6, 53.8) with 100 nM mirabegron and 31% (14.3, 47.2) with 150 nM mirabegron (*p* = 0.0036) after 20 h, compared to means of controls. By trend, increases maintained after 40 h, including increases by 19% (0.6, 37.8) with 50 nM mirabegron, by 19% (1.7, 36.3) with 100 nM mirabegron and 21% (−19.9, 62.3) with 150 nM mirabegron (Figure 4B). No alterations were observed in the presence of L-748,337 (Figure 4C).

### 3.6 Effects of mirabegron on muscarinic receptors

**FIGURE 5:**
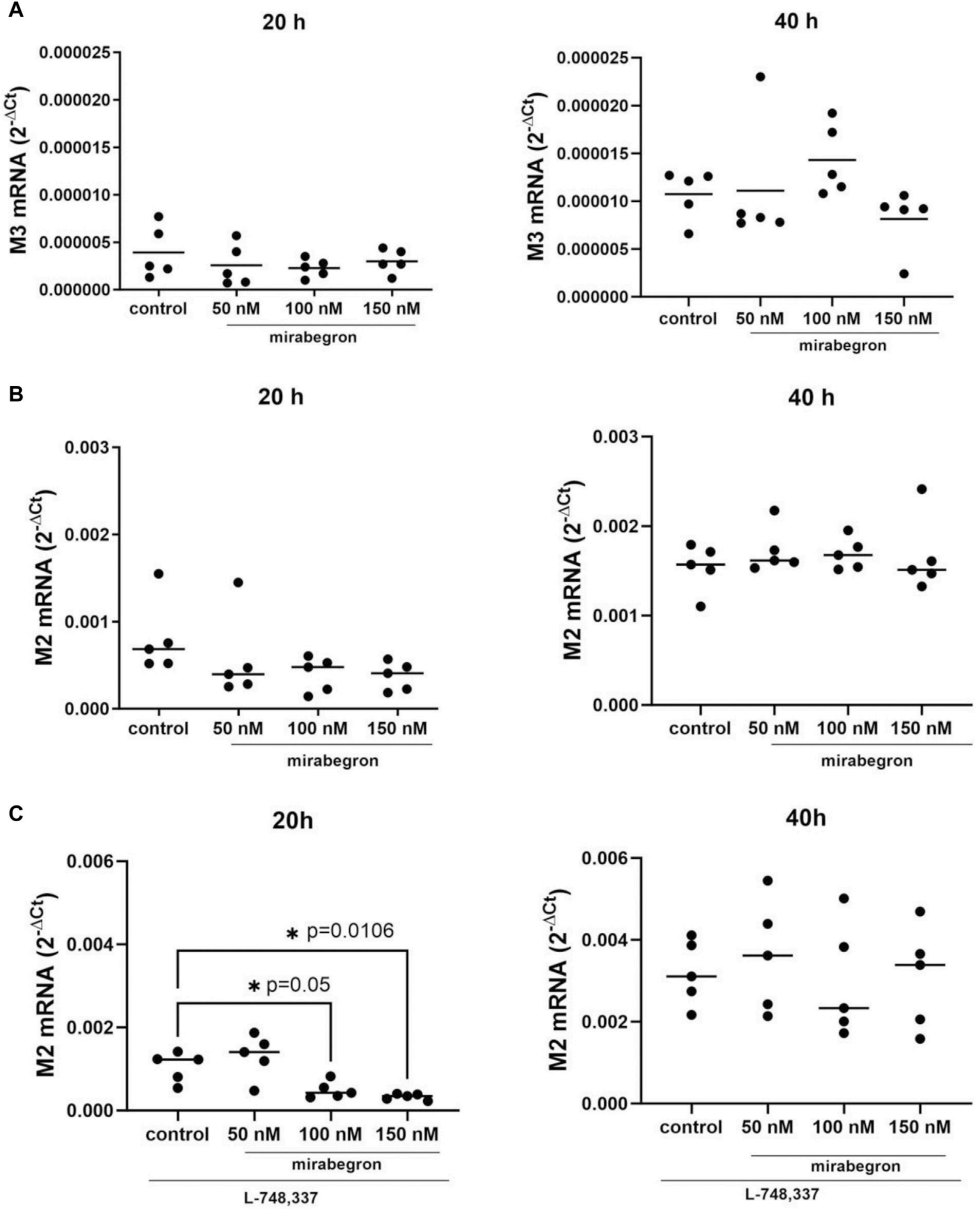
Effects of mirabegron on muscarinic receptors in hBSMC. mRNA expressions of muscarinic M3 receptor **(A)**, muscarinic M2 receptor **(B)**, muscarinic M2 receptor in the presence of L-748,337 (1 µM) **(C)** were determined after 20 h or 40 h of mirabegron exposure or after analog treatment with solvent (controls). Shown are all data from n = 5 independent experiments, in scatter plots with means **(A–C)** and together with *p* values from Kruskal Wallis followed by Dunn’s multiple comparisons test (**A,B**, 20 h; **C**, 40 h) and ordinary one-way ANOVA followed by Dunnett’s multiple comparisons test (**B**, 40 h; **C**, 20 h) (*p < 0.05 vs. control; *p* values ≥ 0.05 not shown).

Expression of M3 mRNA was slightly reduced in mirabegron-treated groups after 20 h. The levels were 0.66 fold (−0.04, 1.4) of mean of controls with 50 nM, 0.58 fold (0.28, 0.89) with 100 nM and 0.77 fold (0.37, 1.17) with 150 nM. Values after 40 h were 1.04 fold (0.26, 1.8) of mean of controls with 50 nM, 1.33 fold (0.91, 1.76) with 100 nM and 0.76 fold (0.38, 1.14) with 150 nM (Figure 5A). M3 mRNA was undetectable in samples including L-748,337, for unknown reasons.

M2 mRNA expression amounted to 0.71 fold (−0.06, 1.4) of mean of controls with 50 nM, 0.49 fold (0.18, 0.8) with 100 nM and 0.46 fold (0.21, 0.72) with 150 nM after 20 h. After 40 h, M2 mRNA expressions were mostly stable with mirabegron (Figure 5B).

In the presence of L-748,337, M2 mRNA expression decreased significantly in groups treated for 20 h with 100 nM and 150 nM mirabegron. Values amounted to 1.3 fold (0.63, 1.88) of mean of controls with 50 nM, 0.47 fold (0.23, 0.71) with 100 nM (*p* = 0.05), and 0.32 fold (0.23, 0.4) with 150 nM (*p* = 0.0106). No decreases were seen after 40 h with mirabegron and L-748,337 (Figure 5C).

### 3.7 Effects of mirabegron on cell contraction in hBSMC

**FIGURE 6:**
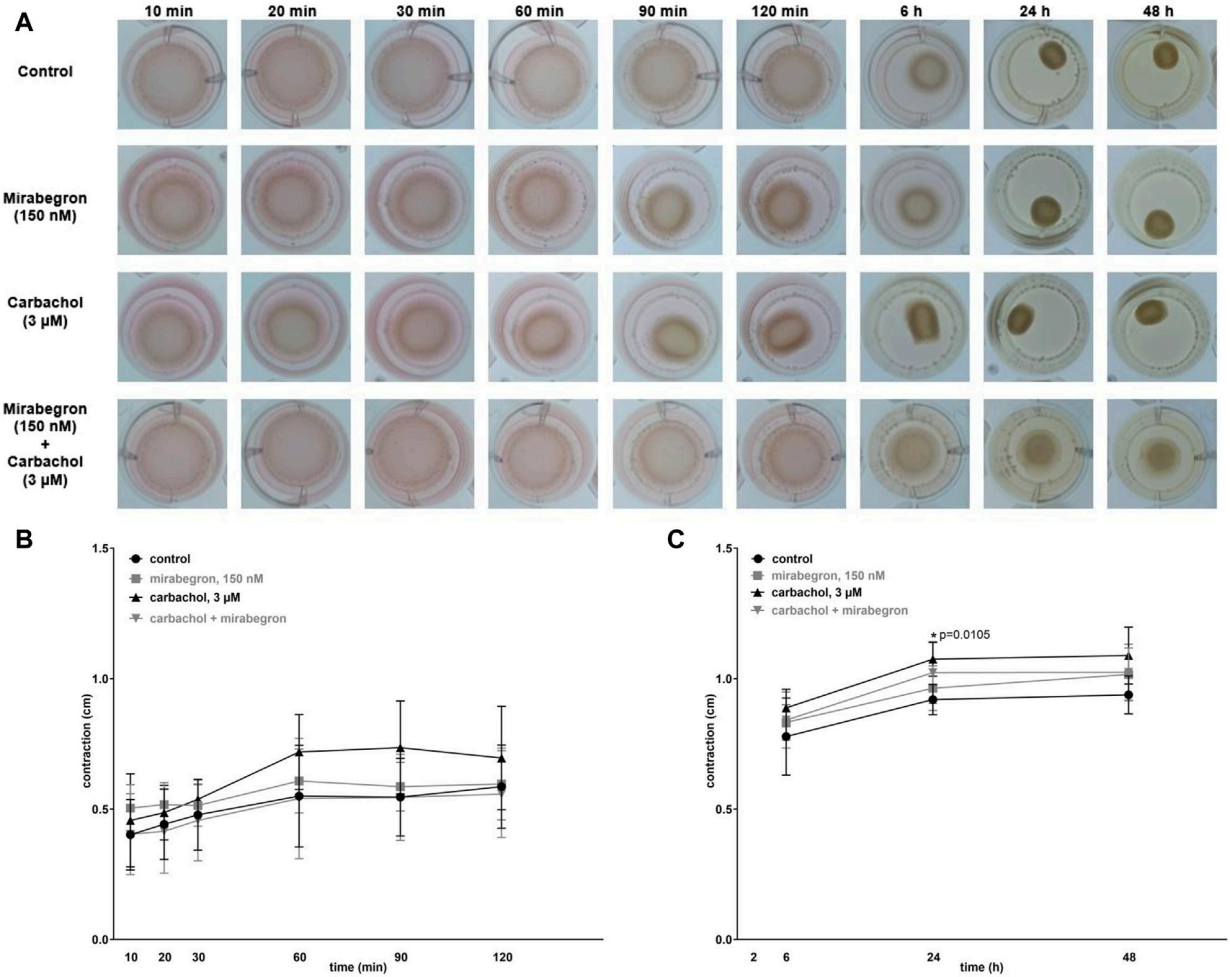
Effects of mirabegron on cell contraction in hBSMC. Contraction of hBSMC was assessed by matrix contraction assays **(A)**, 10–120 min **(B)** and 6–48 h **(C)** after seeding to matrix plugs. Drugs (and solvents for controls) were added with seeding to matrix plugs, as indicated. Contractions are expressed as changes in plug diameter during indicated periods. Shown are all data from n = 5 independent experiments, as means with standard deviation, and together with *p* values from linear regression of two-way ANOVA followed by Dunnett’s multiple comparison test (*p < 0.05 vs. control).

Contraction of hBSMC was assessed by matrix contraction assays, 10–120 min after seeding to matrix plugs, and 6–48 h after seeding in another series (Figure 6A). Contractions increased over time in all groups. Compared to controls, contractions were increased with 150 nM mirabegron by 11% (−17.3, 38.6), 7% (−14.1, 28.4), 2% (−27.9, 30.9) after 60, 90, 120 min respectively, and with 3 µM carbachol by 31% (−1.6, 63.5), 35% (−5.7, 75.1), 19% (−23.3, 60.9) after 60, 90, 120 min respectively (Figure 6B). However, with 3 µM carbachol plus 150 nM mirabegron, contractions were similar to the control group, except of a slight decrease by 5% (−39.7, 30.2) after 120 min (Figure 6B). After 6, 24 and 48 h, contractions were elevated vs. the control group by 14% (2.5, 25.3), 17% (7.8, 25.7) (*p* = 0.0105), 16% (1.4, 30.5) with 3 µM carbachol, and by 7% (−4.2, 17.9), 5% (−6.6, 16.5), 8% (−5.0, 21.6) with 150 nM mirabegron (Figure 6C). In the 3 µM carbachol plus 150 nM mirabegron group, contractions increased by 8% (−9.1, 24.9), 11% (−4.9, 27.4), 9% (−5.2, 23.3) after 6, 24, 48 h, respectively compared to the control group (Figure 6C). By comparison of the control group with all other groups, the increase was only significant for carbachol after 24 h (*p* = 0.0105), but not significant for carbachol plus mirabegron after 24 h (Figure 6C).

## 4 Discussion

Our findings suggest dynamic effects of exposure to mirabegron for up to 48 h on phenotype markers in hBSMC, paralleled by attenuation of muscarinic receptor mRNA and by changes in phenotype-dependent functions. In fact, mechanisms underlying symptom improvements by mirabegron in OAB are unclear, and molecular impacts of exposure exceeding few hours are unknown in bladder cells, as previous *in vitro* studies were to the best of our knowledge limited to acute effects. Effects observed in our study occured with concentrations in the range of known plasma levels, and point to dynamic, phenotype-related changes of hBSMC by mirabegron, including markers for contractile and non-contractile phenotypes of smooth muscle cells. Despite concentration ranges suggesting specificity for β_3_-AR, effects of mirabegron were partly resistant, and partly sensitive to a β_3_-AR antagonist. Together, our observations from long-term exposure of hBSMC to mirabegron may provide new explanations for clinical effects of mirabegron in OAB.

Calponin-1 is specifically expressed in differentiated smooth muscle cells and promotes contractility (Liu and Jin, 2016; Feng et al., 2019). MYH11 abounds in smooth muscle cells including vascular smooth muscles, bladder, intestine and uterus (Yokoyama et al., 2018) and takes part in smooth muscle contraction as well (Li et al., 2018). Four MYH11 isoforms have been identified in human tisssues, including SM1, SM2, SM-A and SM-B (Yokoyama et al., 2018). In our present study, we observed decreases of calponin-1 mRNA expressions with mirabegron after 20 h, which was resistant to β_3_-AR antagonist. Consequently, mirabegron may contribute to relaxation of hBSMC by attenuating the contractile marker calponin-1, even independently from β_3_-adrenergic effects but with known plasma concentrations, and at least within 20 h of exposure. In a previous study, the β_3_-AR agonist BRL37344 decreased the expression of α-smooth muscle actin, which was increased after pathological hydrostatic pressure in hBSMC (Lai et al., 2019). Our current findings are in line with this study, as both suggest reducing effects of β_3_-AR agonists on contractile filaments in hBSMC. Interestingly, shared effects in both studies may not necessarily be attributed to β_3_-AR activation, as the applied concentration of BRL37344 was probably unspecific for β_3_-AR (Lai et al., 2019), and because mirabegron effects on calponin-1 were resistant to L-748,337.

Unlike calponin-1, MYH11 increased after 40 h exposure of mirabegron in our present study. Actin-myosin interactions are the key mechanism for smooth muscle contraction, and both calponin-1 and SM-B myosin (one of the isoforms of MYH11) affect this cross-bridge cycling at different kinetic steps (Arner and Malmqvist, 1998; Babu et al., 2006). Babu et al. reported upregulation of calponin-1 in SM-B myosin null mice (Babu et al., 2006), suggesting that this upregulation may be a compensatory mechanism to maintain cross-bridge cycling in the absence of SM-B. Our findings may confirm this mutual regulation, as we observed an opposing regulation of calponin-1 and MYH11. Thus, upregulation of MYH11, occuring time-shifted to downregulation of calponin-1, may represent a compensatory mechanism to maintain contractility of hBSMC against the reduction of calponin-1 induced by mirabegron. Besides, increases of MYH11 reduced with L-748,337, suggesting a role of β_3_-AR-mediated mechanisms in contractile myosin expression. Maccari et al. reported downregulation of cardiac contractile beta myosin heavy chains (βMHC) in mice hearts by intermittent beta blockade, while continuous inhibition of the adrenergic cascade caused upregulation on βMHC (Maccari et al., 2020). In addition, treatment with the β antagonist metoprolol in idiopathic dilated cardiomyopathyc patients downregulated βMHC expessions (Lowes et al., 2002). Thus, effects of adrenergic signaling on MHC expression in the heart are obvious across these previous studies, while our findings show this relationship for the first time in bladder smooth muscle cells. Similar to our study, these previous studies point to highly time-dynamic patterns of these regulations.

While mRNA expression of MYH10 tended to increase after 40 h mirabegron exposure, vimentin mRNA was reduced with or without L-748,337, reflecting phenotype-associated transitions in hBSMC as well. Vimentin, and MYH10 have been previously considered as markers for synthetic phenotypes of smooth muscle cells, characterized by proliferation and low contractility (Obara et al., 2005; Zhong et al., 2018; Zhang et al., 2021). Previous studies reporting switches from contractile to synthetic phenotypes included vascular smooth muscles (Zhong et al., 2018; Cao et al., 2022) and coronary arterial myocytes from CD38 gene deficient mice (Xu et al., 2015), and upregulation of vimentin during this transition. In another study, overexpression of vimentin did not alter contractile markers in murine bladder smooth muscles (Javed et al., 2020). On the other hand, however, vimentin is required for smooth muscle contraction, and lack of vimentin attenuates contractile responses to mechanical stimuli or to agonist stimulation (Tang, 2008). Our current and previous findings may suggest that patterns of phenotype transitions may differ between hBSMC and vascular SMCs, together pointing to bladder- or organ-specific phenotype plasticity, which merits further investigations. As it is believed that mirabegron has relaxing effects on hBSMC and we found that mRNA levels of calponin-1 and vimentin (which are both essential for contraction) were downregulated by mirabegron, we may conclude that mirabegron reduces contractile responses by attenuating contractile proteins. Actin-dependent mechanisms are obviously not involved, as organization of phalloidin-stained actin filaments was not affected by mirabegron.

In our study, cell viability increased with mirabegron after 20 h and still slightly after 40 h, which was sensitive to a β_3_-AR antagonist. Our findings oppose observations in prostate stromal cells, where a slight reduction of viability was observed with mirabegron, which was considered as negligible (Huang et al., 2021). Relationships between cell viability and adrenergic stimulation were studied in various cell types. In osteocytic MLO-Y4 cells treated with isoprenaline, enhanced cell viability was dependent on β_2_-adrenergic signalling (Yao et al., 2017). In contrast, an adrenergic effect on cell viability was shown in murine luteal cells, by increases with norepinephrine and decreases with isoprenaline, revealing that luteal cell viability is positively affected by α-adrenergic activation (Wang et al., 2016). Although these and other studies (Hennenberg and Michel, 2023) provide insights of adrenergic stimulation on survival of non-muscle cells, evidence for β_3_-adrenergic effects on smooth muscle cell viability is still highly limited.

In our EdU assays, mirabegron did not affect the proliferation rate of hBSMC. Similarly, BRL37344 did not affect cell proliferation under pathological hydrostatic pressure in hBSMC in a previous study (Lai et al., 2019). Another study, however, reported decreases in myocyte size by BRL37344 in the murine heart (Watts et al., 2013). Different from the unchanged proliferation rate, we observed increased expression of Ki67, a proliferation marker, after 40 h of mirabegron exposure, which was inhibited by L-748,337. Interestingly, Ki67 expression increased after 20 h of mirabegron exposure only in the presence of L-748,337, but not with mirabegron alone. Ki67 expression commonly, but not necessarily parallels functional changes in proliferation. Similar to our study, discrepant results for Ki67 expression and proliferation have been previously observed as well, and may be caused by alternative splicing of the N-terminus of Ki67, which may differentially affect proliferation (Schmidt et al., 2004). As mirabegron-induced increases in viability occured despite unchanged proliferation, the enhanced viability was probably attributed to mechanisms other than proliferation. Apart from proliferation, increases in viability may be imparted by suppression of apoptosis or cell death. While our current data do not allow conclusions to that, impacts of mirabegron on apoptosis and cell death may be subject of follow-up studies. Together, β_3_-AR-mediated growth responses may in fact exist in bladder smooth muscle, but they are apparently highly dynamic, and outcomes do not depend on β_3_-AR alone. Further studies are required, preferentially *in vivo* and in knockout models, to understand physiological and net effects of β_3_-AR regulation in detrusor growth and in bladder smooth cell muscle proliferation.

Our findings suggest that mirabegron may downregulate expression of muscarinic receptors in hBSMC after 20 h, especially M2, but possibly also the M3 subtype. In human bladder smooth muscle cells, M3 is the subtype predominantly accounting for cholinergic contractions, e.g., during voiding, even though M2 has a higher abundance (Michel et al., 2023). Obviously, the downregulation of M2 occured independently from β_3_-AR activation, as it was even greater with L-748,337. According to documents submitted for approval to the FDA, mirabegron binds to muscarinic M2 receptors (FDA, 2012). Binding of mirabegron to muscarinic receptors was also observed in rat bladders by radioligand binding assay (Yamada et al., 2021). In both models, however, affinities ranged above 2 μM, which is much higher than the concentration applied by us. Carbachol-precontracted rat bladder strips are relaxed concentration-dependently by mirabegron, but decreases in tensions are in fact small (Yamada et al., 2021). In human detrusor strips, mirabegron did not inhibit the maximum, full contractions by carbachol (Huang et al., 2022). Consequently, previous observations from tissue models exposed for short periods to mirabegron and often using unphysiologically high concentrations were probably insufficient to explain symptom improvements in OAB. Downregulation of muscarinic receptors, and of contractile proteins such as calponin may offer a new explanation for clinical effects of mirabegron. However, these effects appeared to be compensated after 40 h in our model, at least at mRNA level. While detection of M2 protein expression may adequately complete our mRNA findings, the use of antibodies for muscarinic receptors, and for a number of other G protein-coupled receptors has been discouraged, as their (subtype-)specificity was found to be limited (Jositsch et al., 2009; Michel et al., 2009; Pradidarcheep et al., 2009). In fact, we initally tried to detect M3 in our samples. However, no signals at all, or no bands with sizes matching the molecular weight of M3 (66 kDa) were obtained (Supplementary Figure S3). As all detected bands had wrong sizes and were probably unspecific, we refrained from further attempts to detect M2 or M3. At this stage, it appears possible that further contractile proteins, not being included in our study are regulated by mirabegron as well. Moreover, phenotype changes may not necessarily be completed within 48 h, and may continue (also with further proteins) after 40 h, when M2 and calponin are compensated (40 h) and thus, under conditions reflecting chronic treatment of OAB with mirabegron. Thus, follow-up studies may include longer exposure times, and further proteins.

Our contraction assays confirmed, that 150 nM mirabegron may reduce cholinergic contractions of hBSMC, whereas it did not reduce time-dependent contractions without carbachol. The inhibition set in during advanced stages of carbachol-induced contractions, starting around 1 h after contraction initiation and still persisting after 24 h, but was not observed in earlier phases of the contraction. Onset of muscarinic receptor downregulation may not be excluded within this period, and may thus account for the anticontractile effect, even though our earliest data from RT-PCR were obtained after 20 h. While cholinergic detrusor contractions are primarily attributed to M3 (Schneider et al., 2004), M2 may be involved in development of bladder smooth muscle tension as well and even represents the major subtype in the human detrusor (Mansfield et al., 2005; Sellers and Chess-Williams, 2012). The role of M2 receptors in contraction seems to be of indirect character, by inhibiting β-adrenergic relaxation responses (Hegde and Eglen, 1999; Ma et al., 2002; Giglio and Tobin, 2009). Based on our current findings, mirabegron may relax bladder smooth muscle by changes at expression level, including contractile proteins and including phenyotype transitions. These changes are obviously dynamic, and may involve further proteins, not being investigated here. Thus, M2 and calponin downregulation may not necessarily be involved in therapeutic effects, as they were compensated after 40 h, along with the increased MYH11 expression. Contractions in matrix contraction assays are time-dependent, occuring over time even without further interventions. Therefore, and as polymerization of the matrix plug is required, the assay is suitable to investigate cell contractions on time scales of hours or days, whereas agonist-induced contractions of tissues occur within seconds to minutes. Similar to tissue contractions, time-dependent contractions of hBSMC in this assay are enhanced by cholinergic agonists, possibly resembling tonic tissue contractions commonly examined with bladder tissues. Different from tissue contractions, where individual variations in microenvironments (collagen content, extracellular matrix composition, pathologies) may differentially influence contractility, contractions in the matrix assay occur under homogenous conditions, i.e., in a defined collagen matrix. Together, the matrix contraction assay partly, though not fully reflects the situation in tissues, and intracellular signaling pathways may or may not be shared by both models.

Mirabegron is available for treatment of storage symptoms in OAB, where it can be chronically applied. Remarkably, however, available *in vitro* studies were limited to acute effects in ranges of minutes or few hours, not allowing changes on expression levels. Even though our conditions, including application for 20–48 h may not fully reflect chronic treatment *in vivo*, our study may be closer to clinical settings compared to previously investigated acute applications. In view of our findings and as changes may continue beyond 48 h, follow-up studies with longer exposure up to 14 days merit further interest. Similarly, a number of *in vitro* studies used mirabegron concentrations exceeding its plasma levels (167 nM) or its affinity for β_3_-AR (2.5 nM) (Krauwinkel et al., 2012; Tasler et al., 2012). Mirabegron concentrations applied here (50–150 nM) are probably specific for β_3_-AR, as they are lower than affinities for β_1_ (383 nM) or β_2_ (977 nM) (Tasler et al., 2012). Thus, blockade of β_1_ and β_2_ by L-748,337 can not be excluded in our experiments, but involvements of these subtypes seem unlikely with 50–150 nM mirabegron. Finally, β_3_ is the major β-AR subtype in the human bladder, whereas β-adrenergic responses in the rat bladder are mixed β_2_/β_3_-responses and largely mediated by the β_2_-AR in the mouse bladder (Hennenberg and Michel, 2023). Nevertheless, we speculate that effects seen with mirabegron in the presence of L-748,337 may not necessarily be mirabegron effects, i.e., no true β_3_-independent mirabegron effects, but could be attributed to L-748,337, as its concentration was not selective for the β_3_-AR. Whether these effects are attributed to adrenoceptors at all or not, can not be finally estimated on the basis of previous or our current data.

## 5 Conclusion

Mirabegron reduces cholinergic contractile responses in hBSMC, and alters the expressions of contractile proteins and downregulates M2 muscarinic receptor mRNA. These effects may be part of phenotype changes induced by chronic exposure to mirabegron, consistently reflected by loss of contractility and enhanced viability. Effects occured with concentrations in the range of plasma concentrations, and may therefore explain improvements of storage symptoms in OAB.

## Data Availability

The original contributions presented in the study are included in the article/Supplementary Material, further inquiries can be directed to the corresponding author.
