# Supplementary material for: Dynamic phenotypic shifts and M2 receptor downregulation in bladder smooth muscle cells induced by mirabegron
Source: Front Pharmacol. 2024 Jul 24;15:1446831. doi: 10.3389/fphar.2024.1446831 (PMC11303193; doi:10.3389/fphar.2024.1446831)
Supplement: Supplementary file 1 [file DataSheet1.PDF]

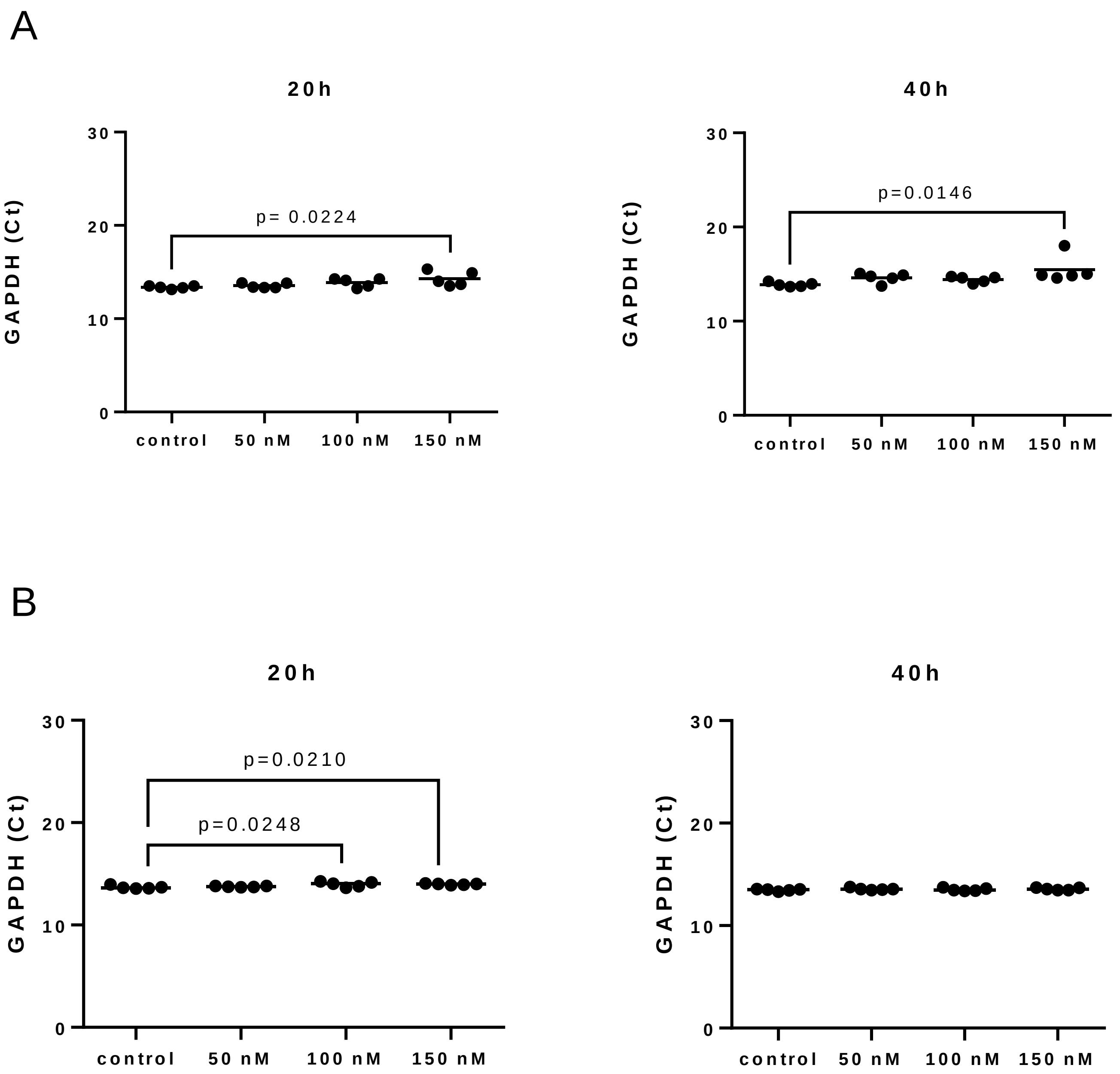

Supplementary figure 1: Ct values (number of cycles) from detection of GAPDH by RT-PCR, after exposure to mirabegron or solvent without L-748,337 **(A)**, or with L-748,337 (1 µM) **(B)**. CNN-1, MYH10, MYH11, VIM and Ki-67 were detected in the same samples, but in separate PCR runs, with each run including de novo detection GAPDH. Consequently, each single value in the diagram represents the mean, from different runs obtained with the same value, from five independent experiments.

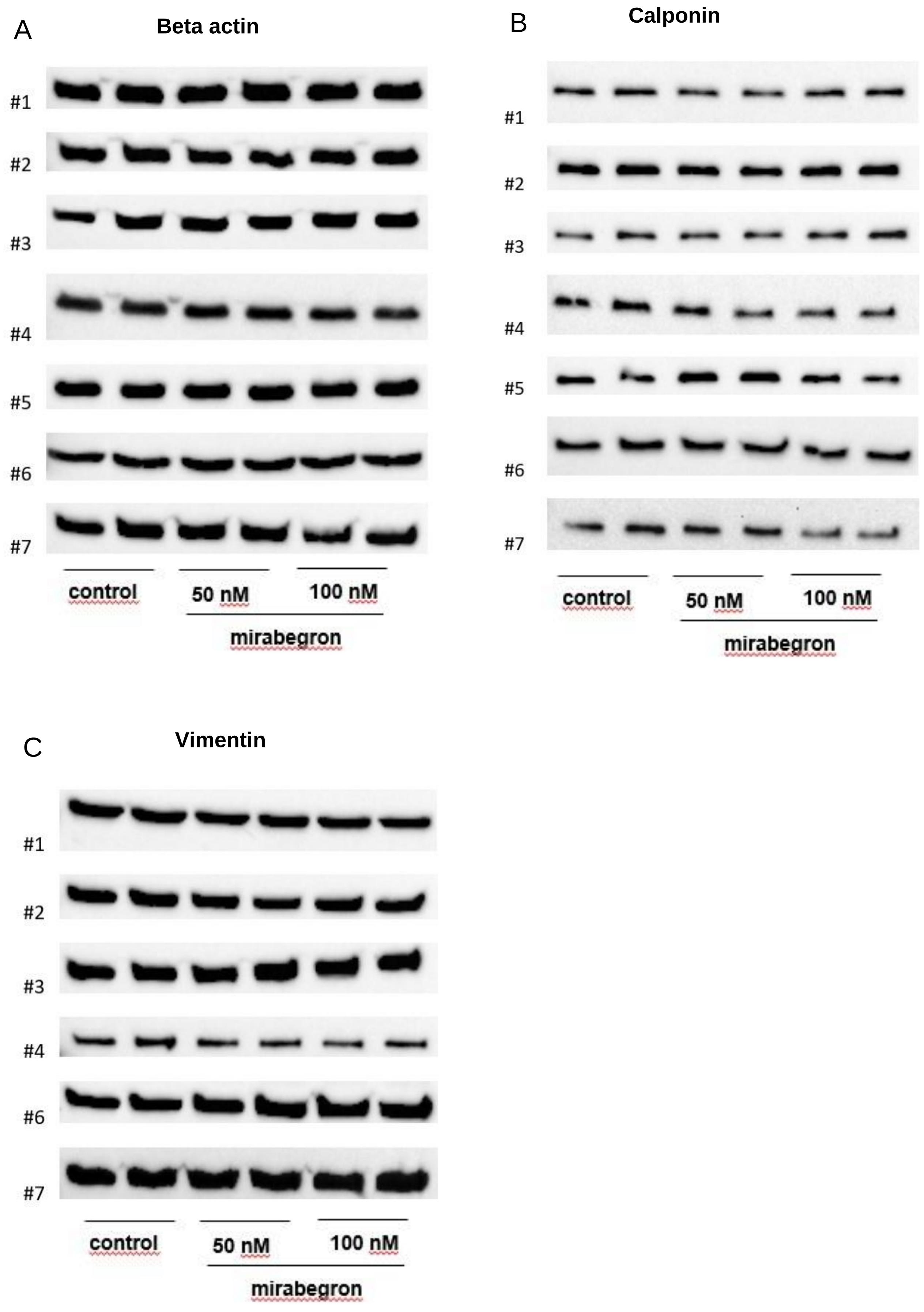

Supplementary figure 2: Blots from each single experiment including Western blot analyses. Detection was performed for β-actin **(A)**, calponin **(B)** and vimentin **(C)**.

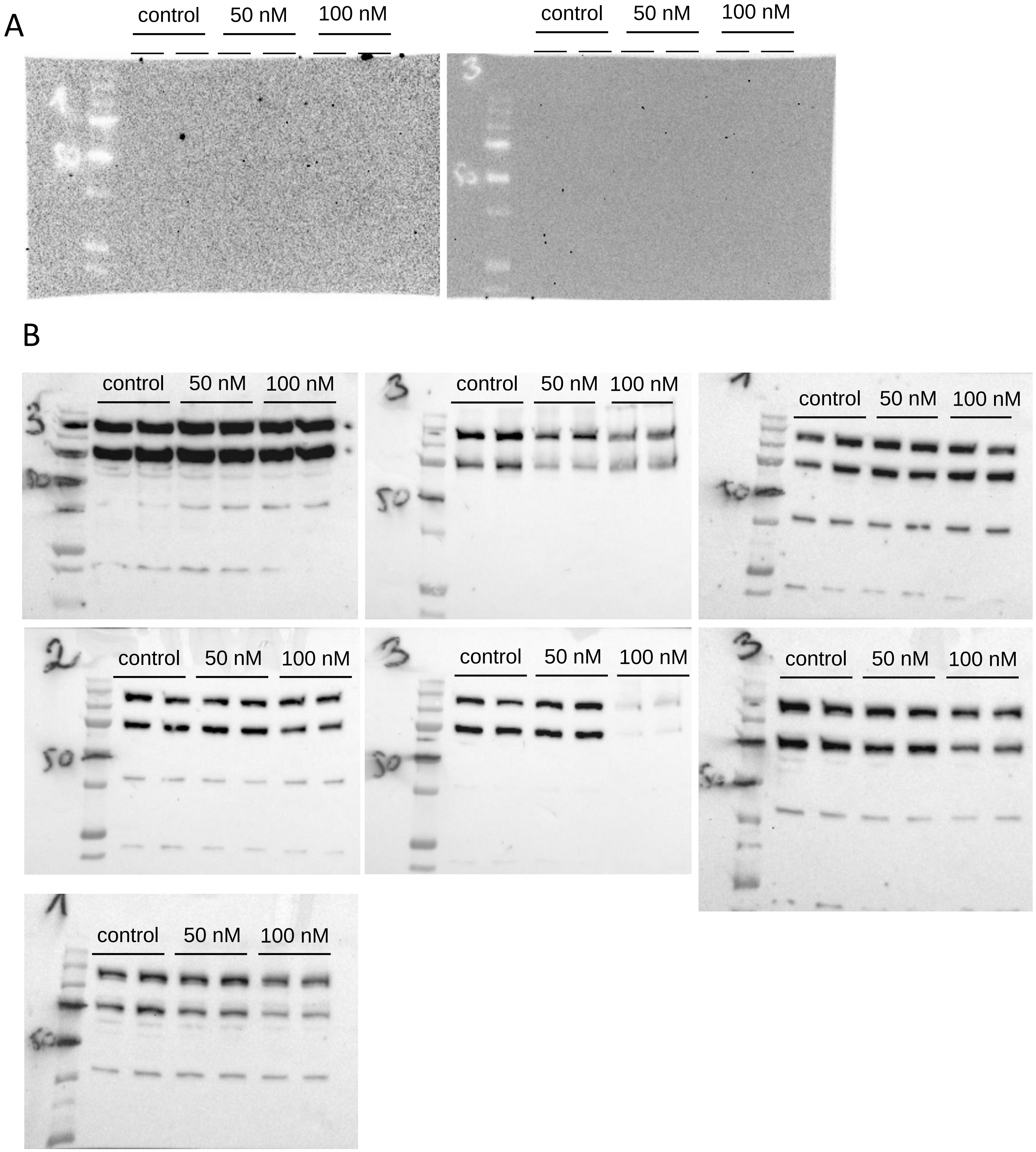

Supplementary figure 3: Western blot analyses of hBSMC (controls, 50 nM mirabegron, 100 nM mirabegron) using a rabbit antibody raised against M3 (MBS1497920, MyBioSource, USA; 1:500), by conventional detection (primary antibody M3, followed by secondary, peroxidase-coupled antibody (as described in materials and methods, with the exception that the secondary antibody was an anti-rabbit) **(A)**, or by amplification using an biotinylated goat anti-rabbit IgG followed by visualization with an avidin/biotin-based peroxidase detection system, exactly as recently described by us (Li et al., FASEB J 2024;38(7):e23604 **(B)**. The molecular weight of M3 is 66 kDa. The 50 kDa band is labelled in each blot, and further bands from molecular weight markers are visible. The next band above the 50 kDa marker has a size of 75 kDa (followed by 100, 150 and 250 kDa). The next band below the 50 kDa marker has a size of 37 kDa (followed by 1-2 strong marker bands of 25 and 20 kDa).
